# mTORC1 inhibitor rapamycin and ER stressor tunicamycin induce differential patterns of ER-mitochondria coupling

**DOI:** 10.1038/srep36394

**Published:** 2016-11-03

**Authors:** Roberto Bravo-Sagua, Camila López-Crisosto, Valentina Parra, Marcelo Rodriguez-Peña, Beverly A. Rothermel, Andrew F.G. Quest, Sergio Lavandero

**Affiliations:** 1 Advanced Center for Chronic Diseases (ACCDiS), Faculty of Chemical and Pharmaceutical Sciences & Faculty of Medicine, University of Chile, Santiago, 8380492, Chile; 2 Institute of Nutrition and Food Technology, University of Chile, Santiago, 7830490, Chile; 3 Cardiology Division, Department of Internal Medicine, University of Texas Southwestern Medical Center, Dallas, Texas, 75235, USA; 4 Center for Molecular Studies of the Cell (CEMC), Faculty of Medicine, University of Chile, Santiago, 8380492, Chile

## Abstract

Efficient mitochondrial Ca^2+^ uptake takes place at contact points between the ER and mitochondria, and represents a key regulator of many cell functions. In a previous study with HeLa cells, we showed that ER-to-mitochondria Ca^2+^ transfer increases during the early phase of ER stress induced by tunicamycin as an adaptive response to stimulate mitochondrial bioenergetics. It remains unknown whether other types of stress signals trigger similar responses. Here we observed that rapamycin, which inhibits the nutrient-sensing complex mTORC1, increased ER-mitochondria coupling in HeLa cells to a similar extent as did tunicamycin. Interestingly, although global responses to both stressors were comparable, there were notable differences in the spatial distribution of such changes. While tunicamycin increased organelle proximity primarily in the perinuclear region, rapamycin increased organelle contacts throughout the entire cell. These differences were paralleled by dissimilar alterations in the distribution of regulatory proteins of the ER-mitochondria interface, heterogeneities in mitochondrial Ca^2+^ uptake, and the formation of domains within the mitochondrial network with varying mitochondrial transmembrane potential. Collectively, these data suggest that while increasing ER-mitochondria coupling appears to represent a general response to cell stress, the intracellular distribution of the associated responses needs to be tailored to meet specific cellular requirements.

Endoplasmic reticulum (ER) and mitochondria are profoundly interdependent organelles^1^,^2^,^3^. Protein folding in the ER, for instance, is a highly energy-demanding process that requires large amounts of ATP, which are provided by mitochondria^4^. In turn, mitochondrial ATP synthesis depends on calcium (Ca^2+^) transfer from ER stores, which stimulates key metabolic enzymes^5^. Localized transfer of ATP and Ca^2+^ requires contact points between both organelles, termed mitochondria-associated ER membranes (MAM). This interface contains proteins like Mitofusin-2 (Mfn2), found both at the ER and mitochondrial surfaces, which acts as a dimeric tethering complex^6^. Phosphofurin acidic cluster sorting protein 2 (PACS2), a multifunctional ER sorting protein, is also important for MAM integrity and composition, as PACS2 knockdown separates mitochondria from the ER^7^. PACS2 maintains MAM enriched with calnexin (CNX), a Ca^2+^-binding chaperone essential for ER protein folding and quality control^8^. However, ER stress induces CNX exit from MAM and relocation to ER quality control domains^9^,^10^. Ca^2+^ transfer at MAM occurs through Ca^2+^–releasing channel inositol trisphosphate (IP_3_) receptor (IP_3_R) at the ER and the mitochondrial voltage-dependent anion channel 1 (VDAC1)^11^. Accordingly, any stimulus leading to IP_3_ production, e.g. histamine, will induce Ca^2+^ transfer from ER to mitochondria.

Although the distance between ER and mitochondria is known to be a key factor that determines the efficiency of Ca^2+^ transfer^12^, it is only recently that the relationship between ER-mitochondria coupling and human pathologies has been recognized and studied. In a previous report from our group, we showed that in the early stage of ER stress, mitochondria and ER increase their contacts, thus enhancing Ca^2+^ transfer and increasing mitochondrial metabolism as part of an adaptive response^13^. Of note, we showed that the increase in ER-mitochondria contacts is observed specifically in the perinuclear area, where the ER quality control machinery accumulates during ER stress conditions^14^,^15^,^16^. These observations suggest a localized increase in energy demand that spatially regulates ER-mitochondria responses. In related work, we went on further to show that a reduction in ER-mitochondria coupling during pathological cardiomyocyte hypertrophy may contribute to the decrease in insulin-stimulated mitochondrial Ca^2+^ uptake observed during pathological cardiac remodeling^17^. Our data are consistent with those reported by Fauconnier *et al.*, who identified alterations in cytoplasmic and mitochondrial Ca^2+^ signalling in response to electrical stimulation of adult cardiomyocytes isolated from obese mice, another pathological condition where the heart develops insulin resistance^18^. Interestingly, obesity has also been shown to disrupt ER Ca^2+^ transport in the liver, contributing to ER stress in this organ^19^.

The ER stress response and autophagy are closely linked processes. Both are increased in response to a common set of cellular stress situations (hypoxia, nutrient starvation, poor protein folding, etc.), and evoke processes that seek to restore cellular proteostasis^20^. Moreover, ER stress can trigger signalling events that activate autophagy. Conversely, deficiency in autophagy can lead to ER stress^20^. Nutrient starvation is a classic stimulus for activating autophagy, which then acts to help restore homeostasis in the face of this cellular stress. Inhibition of the mammalian target of rapamycin complex 1 (mTORC1), a metabolic sensor, is a central signalling nodal point in the starvation response. The mTORC1 inhibitor rapamycin is frequently used to stimulate and model starvation-induced autophagy. Nutrient starvation and/or rapamycin treatment have been shown to induce alterations in mitochondria that preserve, or even increase, mitochondrial function in many different types of cells, including HeLa^21^,^22^,^23^,^24^. A number of mechanisms have been implicated in this adaptive change; however, whether remodelling of the ER-mitochondrial network is a fundamental component of this response to nutrient stress or rapamycin is not known. Moreover, in contrast to ER stress, which leads to a focussed response in the nuclear and perinuclear area^13^,^14^, nutrient stress leads to a more generalized cellular response and how this impacts on the ER-mitochondrial network has not been spatially characterized.

Here, we use HeLa cells to evaluate whether the metabolic response to rapamycin involves changes in coupling between ER and mitochondria and to determine whether these responses have spatial features in common with those triggered during ER stress.

## Results

### Both mTORC1 inhibition and ER stress enhance Ca^2+^-regulated mitochondrial bioenergetics

**Figure 1:**
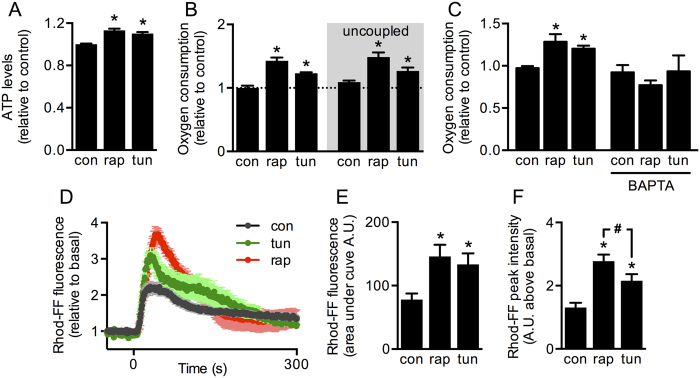
mTORC1 inhibitor rapamycin and ER stressor tunicamycin increase Ca^2+^-regulated mitochondrial bioenergetics. (**A**) ATP levels of control HeLa cells (con) or cells treated with tunicamycin (tun) or rapamycin (rap) were measured using a luciferase-based assay (n = 5). (**B**) Oxygen consumption rates of HeLa cells before and after mitochondrial uncoupling (CCCP 200 nM) were measured using a Clark electrode (n = 5). (**C**) Baseline oxygen consumption rates of HeLa cells in the absence or presence of a Ca^2+^ chelator (BAPTA-AM 20 μM) were measured as in B (n = 4). (**D**) Mitochondrial Ca^2+^ uptake induced by histamine (10 μM) in HeLa cells was measured with Rhod-FF using fluorescence microscopy (n = 3). (**E**) Peak fluorescence intensity of graphs obtained in (**D**). (**F**) Area under the curve of graphs obtained in D. A.U. = arbitrary units. Data are shown as mean ± SEM. *P ≤ 0.05 compared with con, ^#^P ≤ 0.05 comparing tun and rap.

In our previous study, we reported that early stages of ER stress lead to an adaptive increase in mitochondrial bioenergetics, which depends on an increase in ER-to-mitochondrial Ca^2+^ transfer^13^. During a starvation stress response, which may be induced by mTORC1 inhibition, others have reported that preserving mitochondrial metabolism plays a key role in cell survival^21^. To assess whether the response to starvation stress is similar to that triggered during ER stress, we compared the bioenergetics under both conditions in HeLa cells. After mTORC1 inhibition for 4 h with rapamycin (100 nM), total cellular ATP levels increased to a similar extent as observed in response to tunicamycin (0.5 μg/mL, 4 h) induced ER stress (Fig. 1A). The rates of oxygen consumption were also enhanced both in the basal state and after mitochondrial uncoupling with CCCP (200 nM), indicating that mitochondrial metabolism increased in both conditions (Fig. 1B). These increases in cell respiration were prevented by pre-incubation with the cell-permeable Ca^2+^ chelator, BAPTA-AM (20 μM), demonstrating that both responses are Ca^2+^-dependent (Fig. 1C). Similar to the ER stressor tunicamycin, rapamycin increased mitochondrial Ca^2+^ uptake elicited by histamine (10 μM), as measured using the mitochondria-specific Ca^2+^ indicator Rhod-FF (Fig. 1D). Although the magnitude of Ca^2+^ transfer was similar in both conditions (Fig. 1E), we noted that rapamycin induced significantly higher peak values when compared to tunicamycin (Fig. 1F), suggesting more extensive ER-mitochondrial coupling. These differences were not attributable to an increase in total Ca^2+^ release from ER stores, as there was no significant difference in the magnitude of cytoplasmic Ca^2+^ release as measured with the cytosolic Ca^2+^ indicator Fluo-3 (Fig. S1A,B). Changes in mitochondrial mass were also dismissed, as the mitochondrial protein marker mtHSP70 showed no changes when compared with levels of the housekeeping protein GAPDH (Fig. S2A). To confirm that rapamycin and tunicamycin induce stress responses, we evaluated JNK phosphorylation levels and found that they were elevated in both cases (Fig. S2B). Consistent with its role as an mTORC1 inhibitor, only rapamycin reduced the phosphorylation of p70 S6K, a direct target of mTOR (Fig. S2C). Additionally, tunicamycin increased the mRNA levels of the ER chaperone BiP, while rapamycin did not (Fig. S2D), indicating that tunicamycin induces a typical ER stress response, while rapamycin induces a different kind of stress generated by disruption of nutrient sensing. In summary, these results show that enhanced ER-to-mitochondria Ca^2+^ transfer and increased mitochondrial metabolism are common adaptive responses to both ER and starvation stress.

### mTORC1 inhibition and ER stress induce different patterns of ER-mitochondria contacts

**Figure 2:**
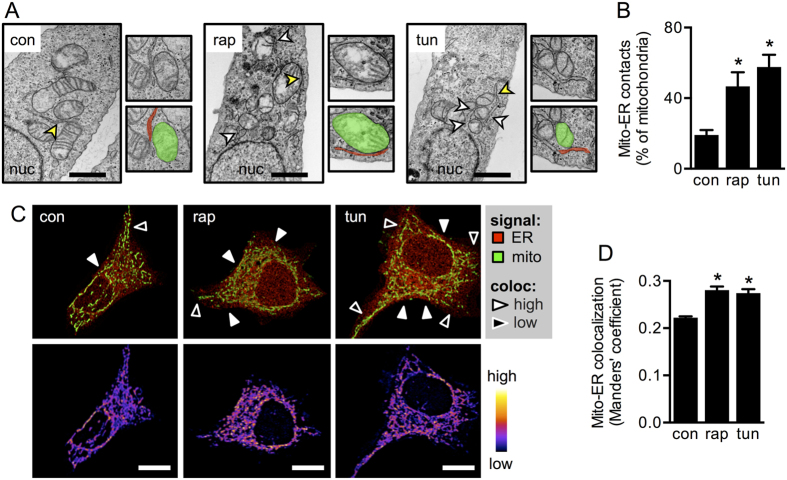
mTORC1 inhibitor rapamycin and ER stressor tunicamycin increase ER-mitochondria contacts. (**A**) Electron micrographs of control HeLa cells (con) or cells treated with tunicamycin (tun) or rapamycin (rap). Arrowheads indicate ER-mitochondria contact sites. Scale bars = 1 μm. Upper insets show magnifications of the area indicated by yellow arrowheads. Lower insets show in pseudocolors mitochondria (green) and ER (red) in the magnified areas. (**B**) Percentage of mitochondria in contact with ER cisternae of cells imaged as in A. (**C**) Upper panels: mitochondria (green) and ER (red) of HeLa cells stained with MitoTracker Green and ER-RFP respectively, measured by confocal fluorescence microscopy. Lower panels: pseudocolor images indicating colocalization of both organelles. Scale bars = 10 μm. (**D**) Mitochondria-to-ER colocalization of images acquired as in (**C**) calculated as the Manders’ coefficient (n = 3). Data are shown as the mean ± SEM. *P ≤ 0.05 compared with controls (con).

Two different methodologies were used to determine whether increased Ca^2+^ transfer correlated with a change in the extent of physical contacts between ER and mitochondria. First, high resolution electron microscopy showed that in these two models of ER and nutritional stress there was an increase in the number of mitochondria juxtapositioned to ER cisternae (Fig. 2A,B). Second, fluorescence confocal microscopy showed that both conditions increased ER-mitochondria proximity (Fig. 2C,D). Previously, we showed that an increase in organelle proximity is required to increase inter-organelle Ca^2+^ transfer observed during ER stress^13^. Taken together the results in Fig. 2 suggest that increased organelle contacts are a common feature that contributes to metabolic adaptation in both the ER and nutritional stress responses.

**Figure 3:**
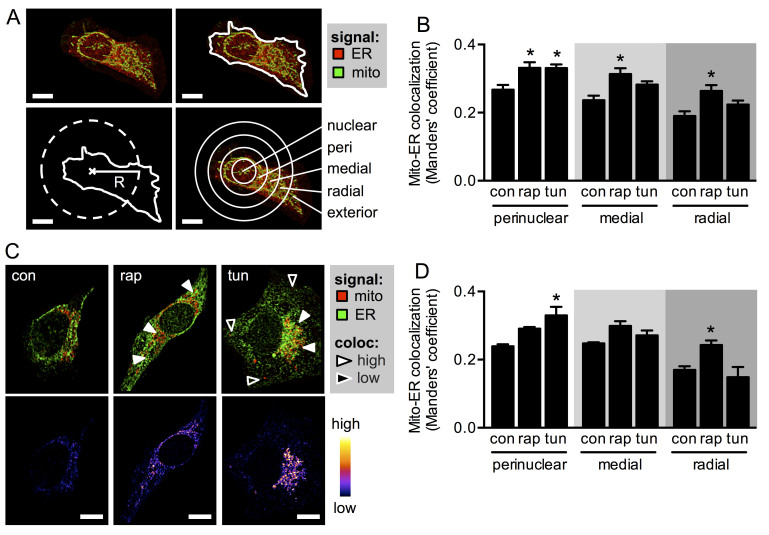
mTORC1 inhibitor rapamycin and ER stressor tunicamycin induce different patterns of ER-mitochondria communication. (**A**) Example of the radial analysis in HeLa cells co-stained with ER-RFP (red) and MitoTracker Green (upper left). After image acquisition, cells were delineated and their area was determined (upper right). From this area, a hypothetical cell radius was calculated (lower left). According to this radius, 4 concentric circles were defined, using the middle of the nucleus as their centre (lower right). Analysis of fluorescence was then performed in the 5 regions defined by the circles. (**B**) Mitochondria-to-ER colocalization of control HeLa cells (con) or cells treated with tunicamycin (tun) or rapamycin (rap), calculated locally as the Manders’ coefficient within regions of the radial analysis (n = 3). (**C**) Upper panels: immunofluorescence of ER (anti-KDEL antibody, green) and mitochondria (mtHsp70, red) of control MDA-MB-231 cells (con) or cells treated with tunicamycin (tun) or rapamycin (rap), measured by confocal fluorescence microscopy. Lower panels: pseudocolor images indicating colocalization of both organelles. (**D**) Mitochondria-to-ER colocalization of images obtained as in (**C**) calculated locally as radial Manders’ coefficients (n = 3). Scale bars = 10 μm. Data are shown as the mean ± SEM. *P ≤ 0.05 compared with controls (con).

Despite the similarities in the stress response to both stimuli, more detailed analysis of the confocal images revealed that tunicamycin and rapamycin induced different patterns of organelle interaction. The increase in ER-mitochondria proximity in response to ER stress was restricted to the perinuclear region of the cells, whereas, rapamycin-mediated mTORC1 inhibition had a more global effect, involving peripheral regions of the cell, in addition to the region around the nucleus (Fig. 2C). To quantify this, we performed a radial analysis of organelle colocalization, as previously described (Fig. 3A)^13^. This method permits spatial analysis of fluorescence with respect to the centre of the cell nucleus, by dividing the protoplasm in 5 concentric rings, termed nuclear, perinuclear, medial, radial and exterior regions. Radial analysis indicated that the ER stressor tunicamycin significantly increased organelle colocalization only in the perinuclear region. In contrast, in rapamycin-treated cells colocalization increased in both the perinuclear region and more peripheral areas (medial and radial regions) that represent the bulk of the cytosol (Fig. 3B). To test whether this is a generalized biological response, we also performed the experiment in another cell line. In HeLa cells, rapamycin has been shown to be a specific inhibitor of mTORC1, but not of mTORC2^25^. Likewise, in MDA-MB-231 cells, rapamycin reportedly shows a similar mTORC1 specificity^26^. In these cells, rapamycin-mediated mTORC1 inhibition (Fig. S3A) lead to increased ER-mitochondria proximity in all regions, but only became significant in the cell periphery, whereas tunicamycin induced changes predominantly in the perinuclear region (Fig. 3C,D, Fig. S3B). With regards to other stimuli, we previously showed that thapsigargin-induced ER stress also leads to localized ER-mitochondria interaction^13^. Similarly, we tested whether other conditions leading to mTORC1 inhibition may alter global organelle interaction. After 4 h of treatment, both amino acid depletion (EBSS medium) and glucose starvation (RPMI medium) led to mTORC1 inhibition (Fig. S4A). Interestingly, while amino acid depletion did not alter ER-mitochondria colocalization, glucose starvation induced a similar response to that observed with rapamycin, namely globally-enhanced ER-mitochondria proximity (Fig. S4B–D). These results show, for the first time using two different types of stress situations that both increase physical coupling between ER and mitochondria, but do so with distinguishable spatial distribution patterns within the cell.

**Figure 4:**
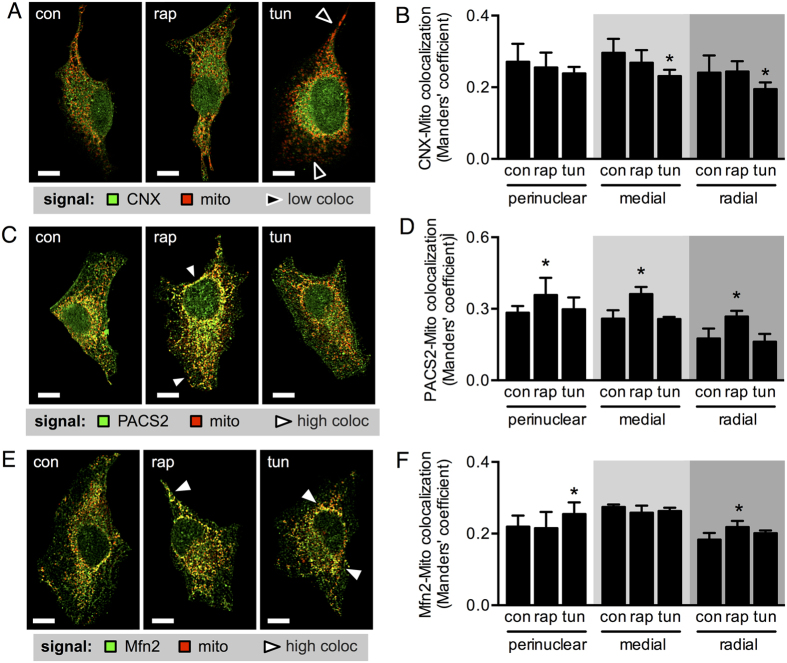
mTORC1 inhibitor rapamycin and ER stressor tunicamycin mobilize different sets of ER-mitochondria connectors. (**A**) Immunofluorescence of CNX (green) and MitoTracker Orange staining (mito, red) of control HeLa cells (con) or cells treated with tunicamycin (tun) or rapamycin (rap), measured by confocal fluorescence microscopy. (**B**) CNX-to-mitochondria colocalization of images acquired as in A, calculated locally as radial Manders’ coefficients (n = 3). (**C**) Immunofluorescence of PACS2 (green) and MitoTracker Orange staining (mito, red) of control HeLa cells (con) or cells treated with tunicamycin (tun) or rapamycin (rap), measured by confocal fluorescence microscopy. (**D**) PACS2-to-mitochondria colocalization of images acquired as in (**C**) calculated locally as radial Manders’ coefficients (n = 3). (**E**) Immunofluorescence of Mfn2 (green) and MitoTracker Orange staining (mito, red) of control HeLa cells (con) or cells treated with tunicamycin (tun) or rapamycin (rap), measured by confocal fluorescence microscopy. (**F**) Mfn2-to-mitochondria colocalization of images acquired as in (**E**) calculated locally as radial Manders’ coefficients (n = 3). Scale bars = 10 μm. Black arrowheads show zones of low colocalization; white arrowheads show zones of high colocalization. Data are shown as the mean ± SEM. *P ≤ 0.05 compared with controls (con).

Among the many proteins that are present in MAM, some of them have the capacity to transit into or out of these microdomains, according to cellular needs^10^,^27^,^28^. For this reason, we predicted that heterogeneities in the distribution of MAM proteins should exist throughout the cell that would account for the spatial differences in ER-mitochondria coupling observed under the two types of stress situations. To test this, we analysed colocalization between mitochondria and two ER-resident proteins normally enriched in MAM. In the case of CNX, which translocates out of MAM upon ER stress, as reported using cell fractionation^9^,^10^, we did not detect changes in global colocalization following either tunicamycin or rapamycin treatment (Fig. S5A). However, spatial analysis revealed a decrease in colocalization in the more peripheral regions with the ER stressor tunicamycin, but not as a consequence of nutritional stress (Fig. 4A,B). This observation cannot be attributed to changes in CNX distribution (Fig. S5B). The fact that rapamycin did not lead to CNX relocation suggests that this process is linked to the specific role of CNX in protein folding rather than a general function in stress responses. Moreover, regions that maintain CNX-mitochondria colocalization correlated with zones where ER-mitochondria contacts increased, thus suggesting that CNX is required to enhance local organelle communication. Global PACS2-mitochondria colocalization, in turn, tended to increase during nutritional stress, but not as a consequence of ER stress (Fig. S5C). Spatial analysis revealed that colocalization increased throughout the entire cell upon rapamycin treatment; however, no such changes were observed with tunicamycin (Fig. 4C,D). Again, this effect was not due to alterations in PACS2 distribution (Fig. S5D). The contrasting observations in both experimental settings support the interpretation that they enhance ER-mitochondria communication through different pathways. Additionally, we also tested Mfn2, which is present in both mitochondria and the ER. For Mfn2-mitochondria colocalization no global changes were detectable in response to either stressor (Fig. S5E). Spatial analysis, however, revealed that tunicamycin increased colocalization only in the perinuclear region, while mTORC1 inhibition increased colocalization only in the radial region (Fig. 4E,F). Mfn2 distribution was not altered in either condition (Fig. S5F). In accordance with our previous study^13^, where Mfn2 ablation abolished the ER-mitochondria adaptive response triggered by tunicamycin, these new results uncover a correlation between perinuclear Mfn2-enrichment in mitochondria and the sites of increased ER-mitochondria interaction. Alternatively, following mTORC1 inhibition, Mfn2 appears to contribute to organelle coupling preferably in peripheral regions. Importantly, the observed changes in protein distribution cannot be attributed to variations in expression levels, as the mass of CNX, PACS2 and Mfn2 remained unchanged for all conditions (Fig. S5G–I).

### Differential profiles of mitochondrial function during mTORC1 inhibition and ER stress

**Figure 5:**
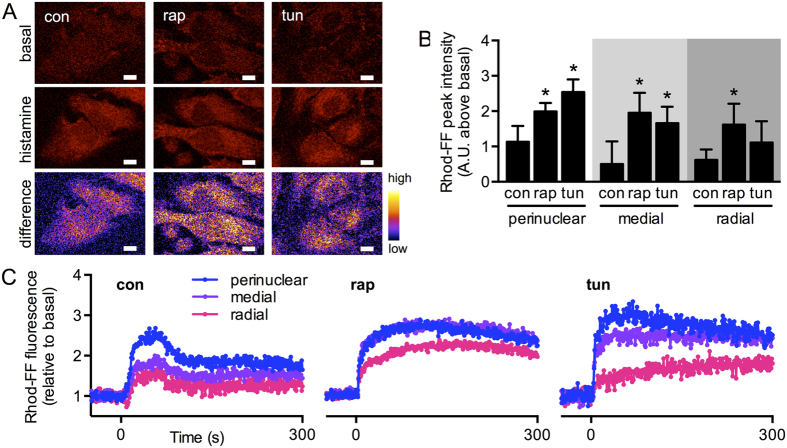
mTORC1 inhibitior rapamycin and ER stressor tunicamycin induce different patterns of ER-mitochondria Ca^2+^ transfer. (**A**) Representative confocal fluorescence images of control HeLa cells (con) or cells treated with tunicamycin (tun) or rapamycin (rap), loaded with Rhod-FF before (upper panels) and after stimulation with histamine 10 μM for 100 s (middle panels). Differences between these images are shown in pseudocolors (lower panels). Scale bars = 10 μm. (**B**) Peak fluorescence intensity of mitochondrial Ca^2+^ signals imaged as in (**A**) calculated locally according to radial analysis (n = 3). (**C**) Representative curves of mitochondrial Ca^2+^ signals imaged as in (**A**) depicting signal differences between regions of the radial analysis. Data are shown as the mean ± SEM. *P ≤ 0.05 compared with controls (con).

To test whether the stress conditions lead to distinctive patterns of mitochondrial Ca^2+^ signalling, we evaluated the spatial distribution of ER-to-mitochondria Ca^2+^ transfer. Quantification of mitochondrial Ca^2+^ images before and after histamine stimulation showed that mitochondrial Ca^2+^ uptake in the control cells occurred preferentially in the perinuclear region (Fig. 5A). In accordance with colocalization results, tunicamycin-mediated ER stress increased the peak intensity of ER-to-mitochondria Ca^2+^ transfer in the more perinuclear regions, while rapamycin treatment induced a global increase in inter-organelle Ca^2+^ signalling (Fig. 5B,C). Again, these spatial differences were not due to local differences in Ca^2+^ levels across the cell, as Ca^2+^ release from the ER remained unchanged in the radial regions (Fig. S1C). Taken together, our results suggest that ER stress and mTORC1-dependent nutritional stress differentially modulate ER-mitochondria coupling, resulting in distinct spatial patterns of Ca^2+^ homeostasis.

**Figure 6:**
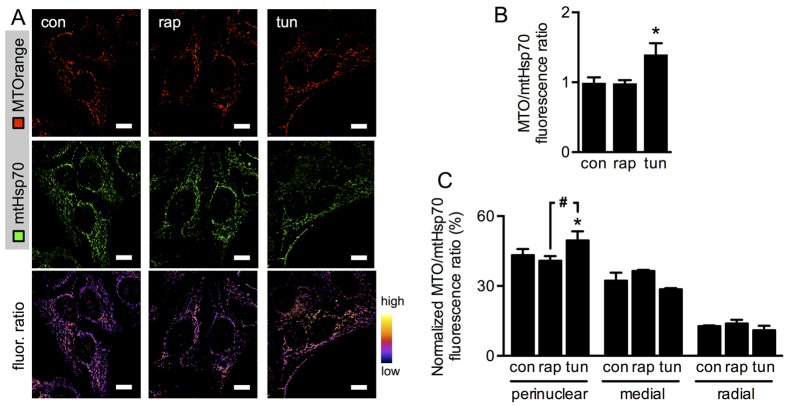
mTORC1 inhibitor rapamycin and ER stressor tunicamycin generate mitochondria with different functional profiles. (**A**) Representative confocal fluorescence images of control HeLa cells (con) or cells treated with tunicamycin (tun) or rapamycin (rap), stained with MitoTracker Orange (upper panels) and mtHsp70 (middle panels). Ratios between these images are shown in pseudocolors (lower panels). Scale bars = 10 μm. (**B**) Global fluorescence ratios between MitoTracker Orange (MTO) and mtHsp70 signals imaged as in (**A**) (n = 3). (**C**) MitoTracker Orange (MTO) and mtHsp70 fluorescence ratios of images obtained as in (**A**) calculated locally according to radial analysis, normalized to the sum of ratios across all regions (n = 3). Data are shown as the mean ± SEM. *P ≤ 0.05 compared with controls (con). ^#^P ≤ 0.05 comparing tunicamycin (tun) and rapamycin (rap).

Mitochondrial transmembrane potential (∆ψ_mt_) is crucial for Ca^2+^ uptake, as it provides the electrochemical gradient that drives Ca^2+^ into the mitochondrial matrix. There, Ca^2+^ stimulates the citric acid cycle, which in turn fuels the generation of ∆ψ_mt_. Furthermore, mitochondrial ATP synthase consumes ∆ψ_mt_ for ATP production. Given these essential roles in mitochondrial function, we analysed the spatial distribution of ∆ψ_mt_ using the probe MitoTracker Orange (MTOrange). As a marker of mitochondrial mass, we used mtHsp70. Interestingly, only the ER stressor tunicamycin increased the MTOrange/mtHsp70 fluorescence ratio (Fig. 6A,B), which is consistent with our previous work^13^. In agreement with our colocalization and Ca^2+^ transfer data, spatial analysis revealed that ∆ψ_mt_ increased significantly during ER stress, and specifically did so in the perinuclear region (Fig. 6C), while mTORC1 inhibition did not induce alterations in ∆ψ_mt_. Altogether, these results suggest that mTORC1 inhibition and ER stress induce different patterns of ER-mitochondria coupling, ultimately leading to distinct mitochondrial activity profiles. On the one hand, ER stress induces heterogeneities in the mitochondrial network, resulting in enhanced perinuclear Ca^2+^ uptake and ∆ψ_mt_ while, mTORC1 preserves mitochondrial homogeneity, increasing Ca^2+^ uptake throughout the whole cell, but without changes in ∆ψ_mt_.

## Discussion

**Figure 7:**
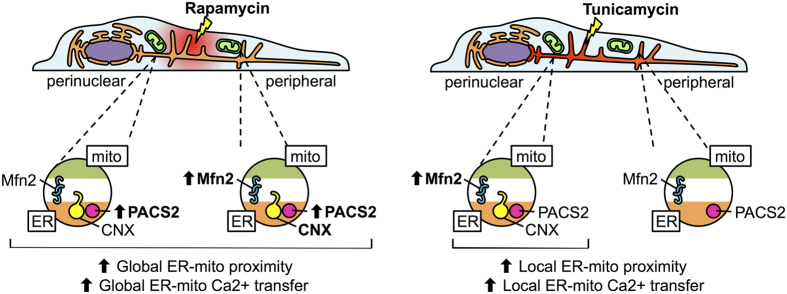
mTORC1 inhibition globally increases ER-mitochondria contacts, while ER stress exerts a localized effect. mTORC1 inhibition mediated by rapamycin induces a global stress response, characterized by an increase in PACS2-mitochondria interaction that leads to a global increase in ER-mitochondria proximity and enhanced mitochondrial Ca^2+^ uptake throughout the whole cytoplasm. ER stress, on the other hand, induces a local response, characterized by a perinuclear increase in Mfn2-mitochondria interaction and a loss of CNX-mitochondria interaction in the cell periphery. This leads to a local increase in ER-mitochondria contacts, concomitant with increased mitochondrial Ca^2+^ uptake and bioenergetics.

The ER-mitochondria junction is an important signalling domain for cell adaptation, especially during conditions that involve metabolic regulation^29^. In this study, we show that an increase in ER-mitochondria contacts and concomitant boost in mitochondrial bioenergetics is a common response for two different types of metabolic stress (Fig. 7). mTORC1 inhibition, on the one hand, is associated with low nutrient supply and insufficient growth factor stimulation (external stress). ER stress, on the other hand, is a condition with high energetic demands in order to restore the protein folding capacity of the ER (internal stress). These data support the notion that modulation of organelle communication represents a generic response to a variety of pathophysiological scenarios. Indeed, disturbances in ER-mitochondria coupling have been associated with various diseases, such as obesity, pulmonary hypertension, insulin resistance and cardiac hypertrophy, among others^29^. However, both responses evaluated here utilize different mechanisms. During mTORC1 inhibition, ER-mitochondria interaction correlated with increased global PACS2-mitochondria colocalization. In contrast, during ER stress, ER-mitochondria interaction correlated with increased Mfn2-mitochondria colocalization in the perinuclear zone and decreased CNX-mitochondria colocalization in more peripheral areas.

The physical association between ER and mitochondria is critical for signalling crosstalk, and ER-to-mitochondria Ca^2+^ transfer is one of the most characteristic functional indicators of organelle coupling^30^. Here, we report that the spatial distribution of ER-mitochondria contacts correlates with the spatiality of Ca^2+^ transfer between both organelles within the cell. This is of particular relevance for Ca^2+^ signalling, which is known to be highly complex and heterogeneous^31^. In this regard, both mitochondria and ER play key roles in modulating Ca^2+^ signals. Mitochondria are highly dynamic structures, which change both their localization within a cell and their network connectivity, thus acting as highly adaptable local Ca^2+^ buffers. Alternatively, the ER is present throughout the cell, but distributed in a non-uniform manner, thereby acting as a heterogeneous Ca^2+^ source. From this perspective, our results show, for the first time, that ER-mitochondria communication is also dynamic at the level of spatial distribution, allowing for different patterns of organelle coupling according to cellular needs.

Aside from being fundamental for energy supply, mitochondria are the guardians of the machinery that initiates programmed cell death^32^,^33^,^34^. Through mitochondrial Ca^2+^ uptake, intracellular Ca^2+^ signalling determines the activity of citric acid cycle dehydrogenases^35^ and directly stimulates the generation of mitochondrial ATP^36^, which is consistent with our findings. However, this stress-dependent increase in mitochondrial Ca^2+^ may act as a double-edged sword, not only occupying Ca^2+^ signalling for cellular adaptation, but also for cell demise in the case of irreparable damage. It should be noted that there are reciprocal effects in this signalling system, as not only does Ca^2+^ regulate mitochondrial function, but also mitochondria shape intracellular Ca^2+^ signals and homeostasis^37^. As complex interconnected organelles, mitochondria form a highly dynamic network governed by the processes of mitochondrial fission and fusion^38^,^39^. In this way, Frieden *et al.* were the first to describe that mitochondrial fragmentation alters the kinetics and propagation of mitochondrial Ca^2+^ transients^40^. Here, we unravel another facet of mitochondrial dynamics by showing that variations in ER-mitochondria interaction can generate either restricted or extensive mitochondrial Ca^2+^ uptake. Whether this modulation contributes to human pathology remains to be determined.

In HeLa cells, rapamycin and starvation induce mitochondrial fusion thus determining the cellular response to autophagy^21^. Although the purpose of mitochondrial elongation during autophagy induction remains unclear, it seems that longer mitochondria are protected from degradation and also possess more cristae and increased ATP synthase activity^21^,^41^. Therefore, dynamic remodelling of mitochondrial shape during rapamycin treatment apparently responds to a global energy demand. As suggested by our results, this increased demand affects the entire cell, as ER-mitochondrial contacts increase throughout the cytoplasm. Remarkably, glucose starvation also induced a global increase in organelle interaction, further supporting this notion. Nevertheless, amino acid depletion did not alter ER-mitochondria colocalization noticeably, thus indicating that other signalling pathways aside from mTORC1 inhibition are required in this case for the increase in organelle communication. On the other hand, it has been shown that the vesicles responsible for autophagic sequestration, termed autophagosomes, form at ER-mitochondria junctions^42^, and thus, the increase in organelle coupling might help facilitate initiation of the phagophore in addition to increasing energy generation. In the case of ER stress, ER-mitochondria contacts form mainly in the perinuclear region, which is the portion of the ER where protein folding takes place^43^. This correlates with our colocalization results, showing that perinuclear CNX-labeled structures remained in contact with metabolically active mitochondria, while colocalization decreased in the cell periphery. Therefore, limited organelle coupling might be associated with localized ATP needs to cope with defective protein folding. Accordingly, after tunicamycin treatment, the observed uncoupling of the ER-mitochondria association or blocking of Ca^2+^ transfer impairs the metabolic response, rendering cells more vulnerable to ER stress^13^. In general, differential cellular distribution of ER-mitochondria coupling might also provide a means to prevent excessive elevations in cytoplasm Ca^2+^, which are known to have deleterious effects on cell survival during stress conditions^2^. In addition to Ca^2+^ homeostasis and energy production, the ER-mitochondria junction carries out multiple functions that are important for cell adaptation. These sites represent signalling platforms for metabolic regulators, such as mTORC2, and participate in the modulation of mitochondrial dynamics, lipid metabolism, and protein trafficking^29^. Further experimentation will be required to elucidate whether these processes are also involved in the ER-mitochondria coordinated response to stress.

Of particular interest are our results related to Mfn2 distribution following rapamycin or tunicamycin treatment, which can be explained by Mfn2 participating not only in mitochondrial fusion, but also in processes such as regulation of ER shape, ER-mitochondria tethering, direct regulation of OXPHOS subunit expression, or simply by the specific expression pattern of Mfn2 in tissues with different metabolic activities^44^. Thus, through the regulation of mitochondrial energy production, Mfn2 may contribute to determining the cell fate, as well as functions that could affect energy expenditure and behaviour, thus explaining our results with rapamycin. On the other hand, our results with tunicamycin showing higher Mfn2-mitochondria colocalization, specifically in the perinuclear area (Fig. 4E,F), are consistent with a more predominant role for Mfn2 in ER-mitochondria tethering. Nonetheless, how cells are able to differentially utilize Mfn2 and take advantage of these distinct functions under a variety of stress conditions requires more detailed studies. All in all, association of mitochondrial quality control, bioenergetics and morphology makes Mfn2 a protein of particular interest for several potential therapeutic approaches in the future.

In summary, we show here that an increase in ER-mitochondria contacts and the Ca^2+^ transfer capacity are part of a general cellular response to different types of cell stress. Interestingly, the spatial distribution of this response can be differentially regulated depending on the stimulus, thereby allowing for greater plasticity in organelle dynamics required to meet local cellular demands.

## Materials and Methods

### Chemicals and cell culture

Reagents were obtained from Merck (Kenilworth, NJ, USA), unless otherwise stated. Tunicamycin was from Enzo Life Sciences (Farmingdale, NY, USA). DMEM, CCCP, rapamycin, histamine and antibody against GAPDH were from Sigma-Aldrich (St Louis, MO, USA). FBS, trypsin/EDTA solution, Lipofectamine 2000, OptiMEM, MitoTracker Green FM, MitoTracker Orange CMTMRos, BAPTA AM, Rhod-FF AM, Fluo-3 AM, antibody against mtHsp70, ECL, TRIzol, reverse transcription reagents, SYBR Green, Earle’s Balanced Salt Solution (EBSS), Roswell Park Memorial Institute (RPMI) 1640 medium and Alexa Fluor conjugated antibodies were obtained from Thermo Fisher Scientific (Waltham, MA, USA). Antibodies against KDEL, CNX, PACS2 and Mfn2 were from Abcam (Cambridge, UK). Antibodies against IP_3_R, p70 S6K, JNK1 and their phosphorylated forms were from Cell Signaling Technologies (Danvers, MA, USA). Cell-Titer Glo Kit was purchased from Promega (Madison, WI, USA). HeLa and MDA-MB-231 cells obtained from ATCC (Manassas, VA, USA) were cultured in Dulbecco’s modified Eagle’s Medium (DMEM) supplemented with 10% fetal bovine serum (FBS), 100 mg/ml streptomycin, and 100 units/ml penicillin in a 5% CO_2_ atmosphere at 37 °C. Cells were plated 24 h prior to experimentation. For amino acid depletion and glucose starvation experiments, EBSS and RPMI media, respectively, were used in the absence of FBS. For transient transfection, cells were seeded in 6-well dishes at 60% confluence and transfected with the ER-targeted RFP construct using OptiMEM and Lipofectamine 2000, following the manufacturer’s specifications.

### ATP determination

ATP determination was performed as previously described^13^. Cells were seeded in 96-well plates at 80% confluence. After treatments, cells were washed twice with PBS. CellTiter-Glo reagent (Promega) was then added, consisting in a luciferin-luciferase solution for the enzymatic determination of ATP by luminometry.

### Oxygraphy

Oxygen consumption experiments were carried out using a previously described method^45^. Cells were seeded in 60 mm dishes at 80% confluence. After treatments, cells were washed with PBS twice, trypsinized for 5 min and centrifuged at 300xg for 5 min. Then, cells were resuspended in PBS and placed in the chamber of a Clark electrode (Strathkelvin Instruments). Oxygen concentration was measured for 3 min to determine the basal respiration of living cells. Then, CCCP (200 nM) was added to determine uncoupled respiration for another 3 min. All rates of oxygen decay were standardized to basal control respiration.

### Live-cell Ca^2+^ microscopy

Intracellular Ca^2+^ levels were assessed using a previously described method^17^. Cells were seeded in 6-well plates on 0.17 mm coverslips at 50% confluence. After treatment, cells were loaded for 30 min with the mitochondrial Ca^2+^ indicator Rhod-FF AM (5.5 μM) and the cytosolic Ca^2+^ probe Fluo-3 AM (4.4 μM). Images were acquired with a Zeiss LSM-5 Pascal 5 Axiovert 200 confocal microscope using a 40x/1.3 objective and an interval of 1 s between images. Baseline fluorescence was recorded for 50 s, then cells were stimulated with histamine (10 μM) and imaged for another 300 s. For each independent experiment, 5–10 cells were imaged, then analysed individually and averaged. For each time point, mean pixel fluorescence background was subtracted and resulting values were standardized to baseline. The area under the curve was calculated during the first 100 s of histamine stimulation.

### Electron microscopy

Electron microscopy experiments were carried out according to a previously described method^13^. Cells were seeded in 100 mm dishes at 80% confluence. After treatments, cells were fixed with 2.5% glutaraldehyde 0.1 M sodium cacodylate, embedded in 2% agarose and post-fixed in buffered 1% osmium tetroxide. En-bloc staining was done with 2% uranyl acetate, dehydration with a graded series of ethanol and embedding in EMbed-812 resin. Thin sections were obtained using a Leica Ultracut UCT ultramicrotome, then stained with 2% uranyl acetate and lead citrate. Images were acquired with a FEI Tecnai G2 Spirit electron microscope equipped with a LaB6 source and operating at 120 kV. ER-mitochondria interaction was quantified as the fraction of mitochondria with in contact with ER cisternae. Approximately 50 mitochondria were analysed per condition.

### Live-cell ER-mitochondria microscopy and colocalization analysis

Live-cell microscopy was performed as previously described^13^. Cells were seeded in 6-well plates on 0.17 mm coverslips at 50% confluence. Cells were transfected with ER-RFP 24 h prior to experimentation, to allow time for protein expression (Fig. S2E). After treatment, cells were loaded for 30 min with the mitochondrial dye MitoTracker Green FM (200 nM). Images were acquired with a Zeiss LSM-5 Pascal 5 Axiovert 200 confocal microscope using a 63x/1.4 objective. For each independent experiment, 5–10 cells were imaged, then analysed individually and averaged. ER and mitochondria images were processed and analysed using ImageJ. First, images were deconvolved using Iterative Deconvolution plugin, then background was subtracted. The default thresholding function of ImageJ was used for image segmentation, i.e. to determine which pixels were background and which pixels could be ascribed to a relevant structure (ER or mitochondria). Colocalization was quantified as the Manders’ coefficient using JACoP plugin for the fraction of mitochondrial pixels that are also labeled an ER structure.

### Immunofluorescence microscopy

Immunofluorescence microscopy was performed as previously described^13^. Cells were seeded in 24-well plates on 0.17 mm coverslips at 50% confluence. After treatment, cells were loaded for 30 min with the mitochondrial dye MitoTracker Orange (400 nM), then fixed for 20 min with 4% paraformaldehyde PBS at 4 °C, permeabilized with 0.1% Triton X-100 PBS for 10 min and blocked with 3% bovine serum albumin PBS for 1 h. Samples were incubated overnight with primary antibodies against KDEL, CNX, PACS2, Mfn2 or mtHsp70 at 4 °C. After incubation with Alexa Fluor 488 secondary antibody for 1 h, samples were mounted with Dako Fluorescence Mounting Medium. Images were acquired with a Zeiss LSM-5 Pascal 5 Axiovert 200 confocal microscope using a 63x/1.4 objective. For each independent experiment, 5–10 cells were imaged, then analysed individually and averaged. For colocalization analysis, images were treated as the aforementioned live-cell ER-mitochondria images. Colocalization was expressed as the Manders’ coefficient, indicating the fraction of CNX, PACS2 or Mfn2 pixels that are also labeled mitochondria. For ratio analysis, images were deconvolved using the Iterative Deconvolution plugin, then background values were subtracted. Finally, values obtained for MitoTracker Orange images were then divided by those from mtHSP70 images.

### Radial analysis

Radial analysis of fluorescence was performed as previously described^13^. Each cell was analysed individually according to their size, as previously published^13^. In brief, from the cell area, a radial distance (r) was calculated using the equation A = π · r^2^. This corresponds to the cell radius, assuming cells were approximately circular (Fig. 3A). Using the centre of the nucleus as a starting point, 4 concentric rings were drawn, defining 5 regions. The nuclear region ranges from 0 to 0.3·r, the perinuclear region from 0.3·r to 0.6·r and encompasses the nuclear envelope and its vicinity. The medial region ranges from 0.6·r to 0.9·r; the radial region from 0.9·r to 1.2·r and includes the cell radius, hence the name. The exterior region (>1.2·r) was excluded from the analysis due to low pixel content. The nuclear region was also excluded, as it was irrelevant for this study. In the remaining 3 regions, fluorescence time lapse, colocalization or ratios were analysed, according to the experiment.

### Immunoblotting

Equal amounts of protein sample were separated by SDS-PAGE (10% polyacrylamide gels) and electrotransferred to nitrocellulose membranes (Bio-Rad, CA, USA). After blocking with 5% milk 0.1% Tween 20 TBS, membranes were immunoblotted with antibodies against phospho-JNK1 (Thr183), JNK1, phospho-p70 S6K (Thr389), p70 S6K, mtHsp70 and GAPDH. Primary antibody binding was detected using peroxidase-conjugated secondary antibodies and ECL, followed by exposure to Kodak film and quantification by scanning densitometry.

### Quantitative real-time PCR

qPCR was carried out as previously described^45^. mRNA samples were obtained using TRIZol reagent, then subjected to reverse transcription to obtain cDNA. SYBR Green was used for real time PCR to detect BiP (primers: forward 5′-TCTGGTGATCAAGATACAGG-3′, reverse 5′-CTTTCACCTTCATAGACCTTG-3′) using HPRT as an internal control (primers: forward 5′-TGCTTTCCTTGGTCAGGCAGTA-3′, reverse 5′-CAACACTTCGTGGGGTCCTTTT-3′). Relative quantities of mRNA were calculated using the Pfaffl’s method^46^.

### Statistical analysis

Results are shown as the mean ± SEM of at least 3 independent experiments. For comparisons among 3 experimental groups, statistical significance (P < 0.05) was assessed using one-way ANOVA followed by Bonferroni post-test. For comparisons of the 3 groups in combination with a stratifying factor (regions of the radial analysis or presence/absence of BAPTA), statistical significance (P < 0.05) was assessed using two-way ANOVA followed by a Holm-Sidak post-test.

## Additional Information

**How to cite this article**: Bravo-Sagua, R. *et al.* mTORC1 inhibitor rapamycin and ER stressor tunicamycin induce differential patterns of ER-mitochondria coupling. *Sci. Rep.* **6**, 36394; doi: 10.1038/srep36394 (2016).

**Publisher’s note**: Springer Nature remains neutral with regard to jurisdictional claims in published maps and institutional affiliations.

## Supplementary Material

Supplementary Information
