# Supplementary material for: mTORC1 inhibitor rapamycin and ER stressor tunicamycin induce differential patterns of ER-mitochondria coupling
Source: Sci Rep. 2016 Nov 3;6:36394. doi: 10.1038/srep36394 (PMC5093439; doi:10.1038/srep36394)
Supplement: Supplementary Information [file srep36394-s1.pdf]

SREP-16-24114R1

Supplementary Material

mTORC1 inhibitor rapamycin and ER stressor tunicamycin induce differential patterns of ER-mitochondria coupling

Roberto Bravo-Sagua[1,2,+], Camila López-Crisosto[1,+], Valentina Parra[1,4], Marcelo Rodríguez-Peña[1], Beverly A. Rothermel[4], Andrew F.G. Quest[1,3,*], and Sergio Lavandero[1,3,4,*]

[1] Advanced Center for Chronic Diseases (ACCDiS), Faculty of Chemical and Pharmaceutical Sciences & Faculty of Medicine, University of Chile, Santiago 8380492, Chile
[2] Institute of Nutrition and Food Technology, University of Chile, Santiago 7830490, Chile
[3] Center for Molecular Studies of the Cell (CEMC), Faculty of Medicine, University of Chile, Santiago 8380492, Chile
[4] Cardiology Division, Department of Internal Medicine, University of Texas Southwestern Medical Center, Dallas, Texas 75235, USA

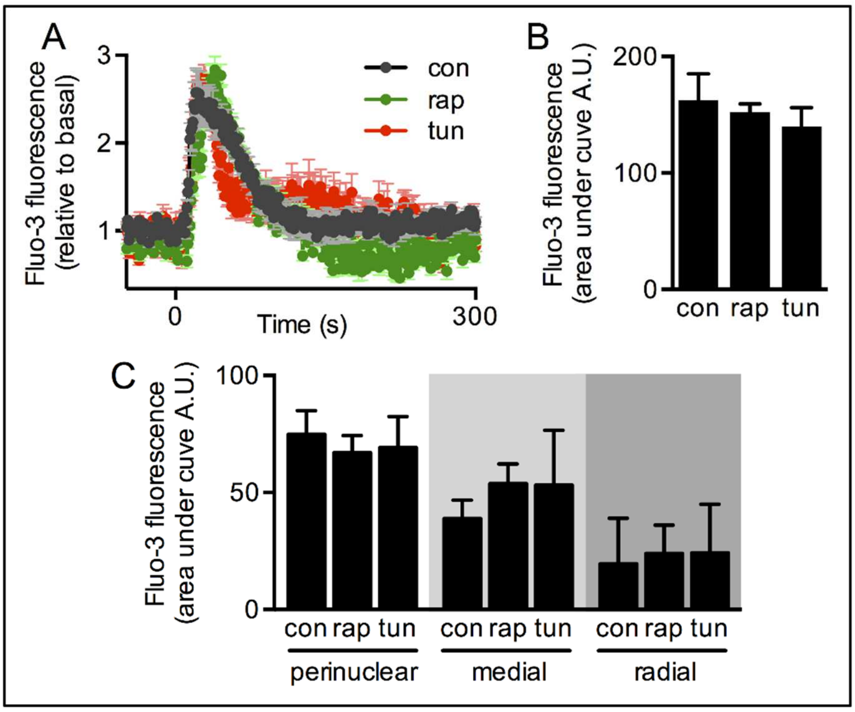

Figure S1. Changes in ER-to-mitochondria Ca^{2+} transfer triggered by rapamycin and tuncamycin are not due to alterations in total Ca^{2+} release. (A) Cytosolic Ca^{2+} release elicited by histamine (10 µM) in control HeLa cells (con) or treated with tunicamycin (tun) or rapamycin (rap) measured with Fluo-3 using fluorescence microscopy (n = 3). (B) Area under the curve of cytosolic Ca^{2+} signals acquired as in A. (C) Area under the curve of cytosolic Ca^{2+} release acquired as in A, calculated locally according to radial analysis (n = 3). A.U. = arbitrary units. Data are shown as the mean ± SEM. Differences are not statistically significant.

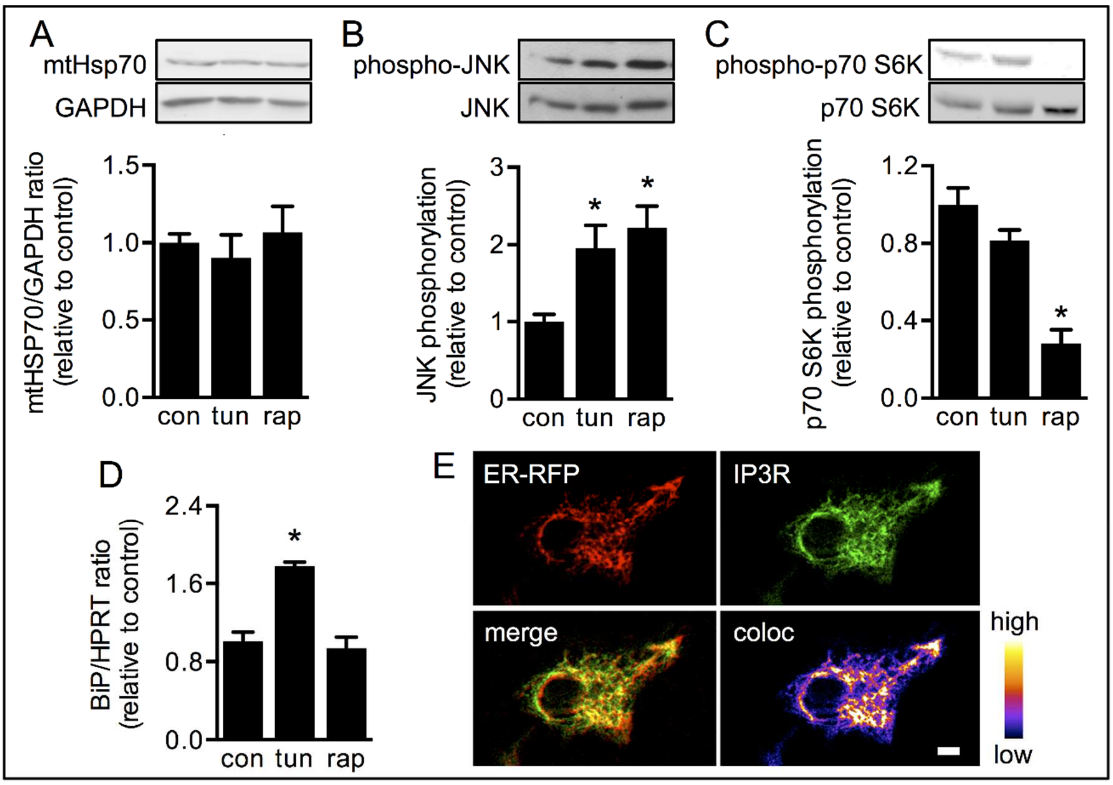

Figure S2. Rapamycin inhibits mTOR, while tunicamycin induces ER stress. (A) Relative protein levels of mtHsp70 and GAPDH in control HeLa cells (con) or cells treated with tunicamycin (tun) or rapamycin (rap), obtained by immunoblot analysis (n = 3). (B) Relative protein levels of phospho-JNK1 (Thr183) and total JNK1 of samples obtained as in A (n = 6). (C) Relative protein levels of phospho-p70 S6K (Thr389) and total p70 S6K of samples obtained as in A (n = 3). (D) Relative mRNA levels of BiP and HPRT of samples treated as in A, obtained by qPCR (n = 3). (E) Representative image of HeLa cells transfected with ER-targeted RFP (upper left), immunostained for the ER marker IP_3R (upper right). Fluorescence merge (lower left) and colocalization (coloc, lower right) show a strong correlation between both signals. Scale bar = 10 µm. Data are shown as the mean ± SEM. *P ≤ 0.05 compared with controls (con).

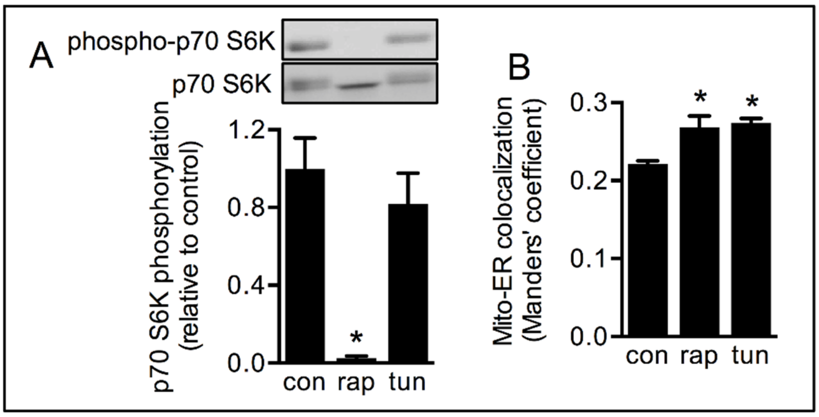

Figure S3. mTORC1 inhibitor rapamycin and ER stressor tunicamycin increase ER-mitochondria communication in MDA-M-231 cells. (A) Relative protein levels of phospho-p70 S6K (Thr389) and total p70 S6K in control MDA-MB-231 cells (con) or cells treated with tunicamycin (tun) or rapamycin (rap), obtained by immunoblot analysis (n = 3). (B) Global mitochondria-to-ER colocalization of MDA-MB-231 cells (con) or cells treated with tunicamycin (tun) or rapamycin (rap), immunostained for ER (anti-KDEL antibody, green) and mitochondria (mtHsp70, red) evaluated by confocal fluorescence microscopy. Scale bars = 10 µm. Data are shown as the mean ± SEM. *P ≤ 0.05 compared with controls (con).

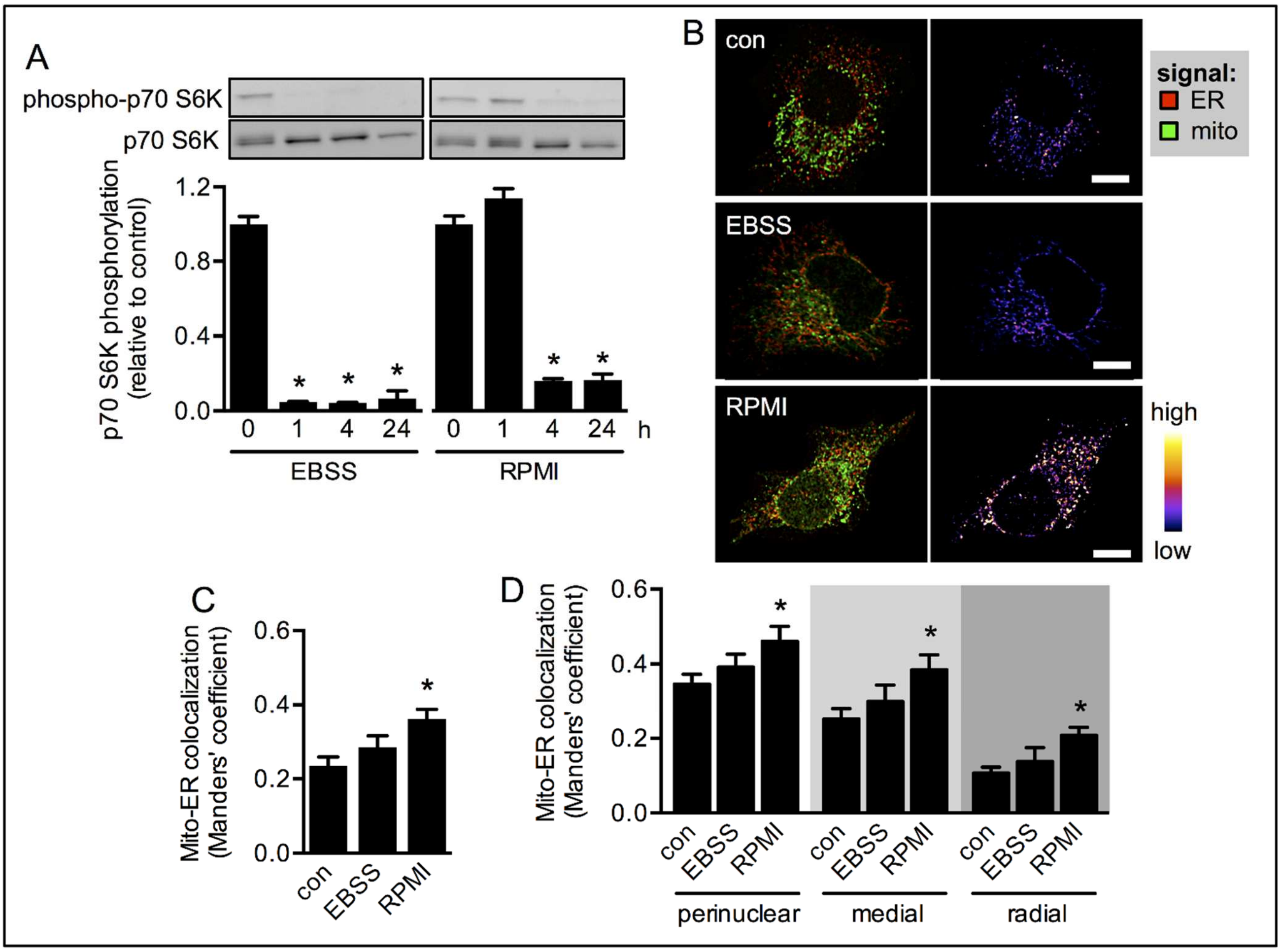

Figure S4. Glucose starvation, but not amino acid depletion, induces a global increase of ER-mitochondria colocalization. (A) Relative protein levels of phospho-p70 S6K (Thr389) and total p70 S6K in control HeLa cells (con) or cells cultured in EBSS or RPMI medium for 1, 4 or 24 h, obtained by immunoblot analysis (n = 4). (B) Left panels: immunofluorescence of ER (anti-KDEL antibody, green) and mitochondria (mtHsp70, red) of control HeLa cells (con) or cells cultured in EBSS or RPMI medium for 4 h, evaluated by confocal fluorescence microscopy. Right panels: pseudocolor images indicating colocalization of both organelles. Scale bars = 10 µm. (C) Global mitochondria-to-ER colocalization of images acquired as in B calculated as the Manders' coefficient (n = 4). (D) Mitochondria-to-ER colocalization of images obtained as in B, calculated locally as radial Manders' coefficients (n = 4). Data are shown as the mean ± SEM. *P ≤ 0.05 compared with controls (con).

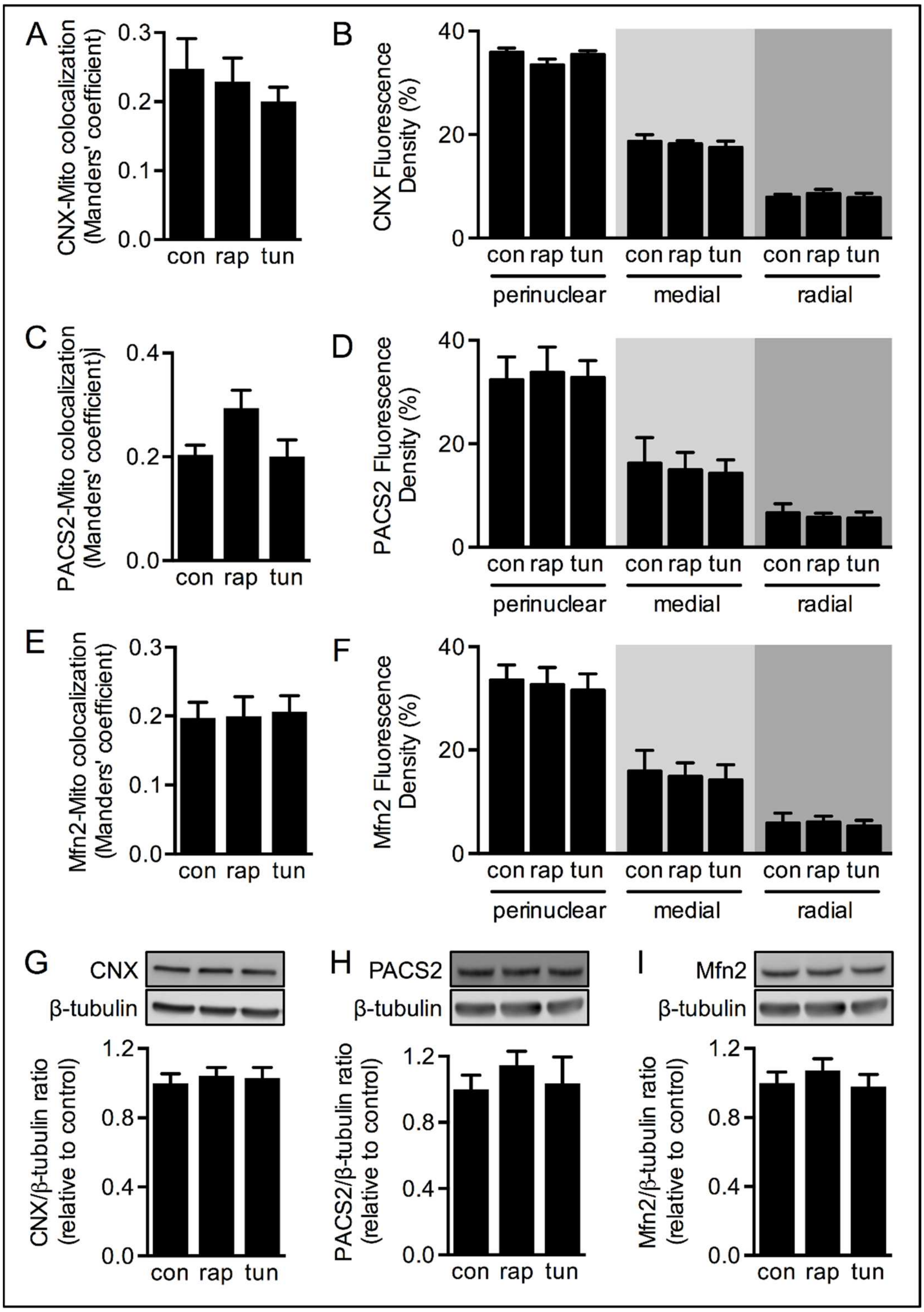

Figure S5. Global colocalization and distribution of MAM proteins in cells treated with rapamycin and tunicamycin. (A) Global CNX-to-mitochondria colocalization in control HeLa cells (con) or cells treated with tunicamycin (tun) or rapamycin (rap), measured by confocal fluorescence microscopy and calculated as the Manders' coefficient (n = 3). (B) CNX fluorescence density in cells acquired as in A,

calculated in the regions of the radial analysis, normalized to total fluorescence (n = 3). (C) Global PACS2-to-mitochondria colocalization in samples treated as in A (n = 3). (D) PACS2 fluorescence density in cells acquired as in C, calculated in the regions of the radial analysis, normalized to total fluorescence (n = 3). (E) Global Mfn2-to-mitochondria colocalization in samples treated as in A (n = 3). (F) Mfn2 fluorescence density in cells acquired as in E, calculated in the regions of the radial analysis, normalized to total fluorescence (n = 3). (G) Relative protein levels of CNX in control HeLa cells (con) or cells treated with tunicamycin (tun) or rapamycin (rap), measured by immunoblot analysis using β-tubulin as loading control (n = 6). (H) Relative protein levels of PACS2 in samples obtained as in G (n = 6). (I) Relative protein levels of Mfn2 of samples obtained as in G (n = 6). Data are shown as mean ± SEM. *$P \leq 0.05$ compared with con.
